# Equitable Access to Health Professional Training in Uganda: A Cross Sectional Study

**DOI:** 10.29024/aogh.7

**Published:** 2018-04-30

**Authors:** M. Galukande, S. Maling, J. Kabakyenga, J. Nshaho, H. Oboke, B. Oonge, H. Muyenje, G. Katumba-Sentongo, H. Mayanja-Kizza, N.K. Sewankambo

**Affiliations:** 1 Department of Surgery, Makerere University, College of Health Sciences, Kampala, UG; 2 Department of Psychiatry, Mbarara University of Science and Technology, Mbarara, UG; 3 Maternal Newborn and Child Health Institute, Mbarara University of Science and Technology, Mbarara, UG; 4 School of Postgraduate Training and Research, Kampala International University, Western Campus, Ishaka, UG; 5 Department of Psychiatry, Gulu University, Gulu, UG; 6 Faculty of Clinical Medicine, Kampala International University, Western Campus, Ishaka, UG; 7 Information Translation Unit, Makerere University, College of Health Sciences, Kampala, UG; 8 Registrar Department, Uganda Medical and Dental Practitioners Council, Kampala, UG; 9 Department of Medicine, Makerere University, College of Health Sciences, Kampala, UG

## Abstract

**Objective:**

We set out to assess inequalities to access health professional education, and the impact of an education improvement program supported by MEPI (Medical Education Partnership Initiative). Inequalities in the higher education system in sub-Saharan Africa remain despite some transformative policies and affirmative action.

**Methods:**

We reviewed enrollment data from four universities for the period 2001–2014 for various health professional training programs, and conducted group discussions through an iterative process with selected stakeholders, and including a group of education experts. Two time periods, 2001–2010 and 2011–2014, were considered. In 2010–11, the MEPI education program began. Gender ratios, regional representation, secondary schools, and the number of admissions by university and year were analysed. We used SPSS version 17 software to analyse these data with level of significance p < 0.05. We collated qualitative data along predetermined and emerging themes.

**Results:**

The overall male-to-female ratio among the student population was 2.3:1. In total, there were 7,023 admissions, 4,403 between 2001–2010 (440 per annum) and 2,620 between 2011–2014 (655 per annum) with p = 0.018. There were no significant increases in admissions in the central and western regions over the two time periods, 1,708 to 849 and 1,113 to 867 respectively, both p = 0.713 and p = 0.253. We propose improving the university admission criteria and increasing enrollment to health professions training schools.

**Conclusion:**

There were significant inequalities for higher education training in Uganda by gender, regional representation and school attended. Modifying the admission criteria and increasing enrollment may reduce these inequalities.

## Introduction

Admissions to medical schools in Uganda and the African region are reported to be predominantly taken up by students from urban areas and from mostly wealthy families [1,2]. This raises questions about equitable access to training opportunities in the health science domain. Equality aims to ensure that everyone gets the same things in order to enjoy full, healthy lives. Equity, like equality, aims to promote fairness and justice, but it can only work if everyone starts from the same place and needs the same things; equity ensures that disadvantages are offset. The eventual employment and deployment of health professionals in underserved areas is likely to be affected by where the graduates trained and by their backgrounds and/or whether they originate from rural, urban, wealthy or disadvantaged families [2,3]. The factors considered before accepting for rural deployment by graduates are factors that affect the quality of life of these graduates in underserved rural areas (good quality schools for children, presence of electricity, running water, access to the internet, television, brain drain, etc.) [4].

The strategies that may improve rural deployment include, but are not limited to, centrally managed postings by the Ministry of Health [5,6]. In addition, the criteria for admissions to medical schools should not be based entirely on academic grades but may also be quota based to achieve an appropriate balance between students from rural and urban backgrounds. There may be complexities in determining the student’s place of origin or background. For example, students may study in rural schools, which are not necessarily poor schools, yet reside in urban centers, and vice versa.

### Overview of higher education in Uganda

Uganda’s secondary education system is composed of two levels of the Ordinary and Advanced. It follows the education system set up by its former colonial masters, the United Kingdom. Lower secondary (ordinary) consists of four years of schooling, at the end of which students undertake ordinary-level exams (O-level) in at least eight subjects with a maximum of 10. Upper secondary (advanced) consists of two years of schooling, at the end of which students sit Advanced-level exams (A-level) in at least three principal subjects [2,7]. Of the 2,564 secondary schools in Uganda, 1,004 are government owned and 1,560 privately owned. Over the past decade, efforts have been made to improve equitable access at secondary school level. For example, these efforts include preferentially equipping predominantly girls’ schools, recruiting teachers and equipping laboratories for rural schools, as well as increasing teacher wages and work conditions [8].

Although 60,000 to 70,000 students in Uganda leave secondary school each year and qualify to go on to higher education, only 35% of them (about 25,000) are able to find places in the few institutions of higher learning [9,10]. As of 2012, Makerere University in Kampala hosted about 95% of the total student population in Uganda’s universities. The remaining students were distributed among five public universities, 27 private universities and a smaller proportion of non-university institutions. The government in 2014 sponsored over 4,000 students offering different courses in public universities. There is no data in the literature stating the proportion of health professions’ trainees taken by Makerere University compared to the rest. The majority (80%) of Uganda’s population and patients reside in rural areas. However, the majority of health workers (especially graduates) are based in urban areas, thus contributing to the rural-urban imbalance in health service delivery and the subsequent unfavorable health outcomes [11,12,13]. Uganda demographic data suggests the majority of secondary schools that succeed in having many of their students enroll for health professions training are expensive, are found in urban areas and are mostly afforded by children from wealthier families. The rural-based primary and secondary schools provide lower quality education; they are fairly affordable and mostly enroll students from poorer families [11]. The students in rural schools score poorer academic grades and may not be admissible to highly competitive health professional courses. To address this challenge there have been increasing calls for changes in the admissions policy [8,14]. However, policymakers have lacked well-documented evidence stretching over several years confirming the existence of this inequity and its magnitude.

The purpose of this study therefore was to document the existence of inequalities in student admissions, propose corrective strategies, and provide baseline data prior to implementation of corrective interventions.

## Methods

### Design

A mixed-method, qualitative and quantitative descriptive study.

### Setting and sampling

This study included the Medical Education for Equitable Services to all Ugandans (MESAU) consortium schools at Makerere University College of Health Sciences, Gulu University, Mbarara University of Science and Technology and Kampala International University (KIU) in Uganda. Since KIU is a private University in the MESAU consortium it did not utilize the Public Universities Joint Admissions Board (PUJAB). It therefore utilizes its own admission criteria though it also based on academic grades and it has a diploma track and mature entry tracks. The MESAU consortium was supported by a Medical Education Partnership Initiative (MEPI) grant meant to improve training of Health workers (quantity and quality) [9].

We collected information available from the admission records of all the universities in the consortium spanning fourteen years (2001 to 2014). Qualitative data were collected from various stakeholders (cross-sectional design). We generated a list of key officials representing relevant institutions which directly related to higher education training and employment for health professionals in Uganda, including the Ministries of Education and Sports, Health, and Public Service. In addition, we included the deans and academic registrars of the four universities and representatives from the different professional councils: Medical and Dental, Allied Professions and Nursing and Pharmacy Councils. We also included student representatives from the five universities and some prominent private practitioners in the Health Sector.

### Data collection and analysis

For the quantitative component, a pre-coded questionnaire was used to extract data from student admission logs and files availed by the Joint Admissions Board (JAB) for undergraduate students enrolled in health professional programs in Medicine, Dentistry, Pharmacy, Nursing, Dental Laboratory, Language and Speech Therapy, Radiography, Public Health Nursing and Environmental Health. Variables included: age, gender, and high-school district and region of origin. In the qualitative study component an interactive process was engaged in with the invited stakeholders and a working group of senior members from the academia (all universities represented). The facilitators introduced the purpose of the meeting. We randomly distributed participants to five groups of 10–12 members each. We assigned guide questions (**Box 1**) that were discussed in each group. After an hour and a half of discussions, we convened a plenary and each of the group presented a summary of their discussions. All the presentations and discussions were recorded with the help of two rapporteurs. At the end of the session we agreed on key points and strategies. We compiled the final framework document after engaging in several meetings and email exchanges over a period of several weeks to reflect the concepts and ideas in the discussions.

Quantitative data were entered and analyzed using SPSS version 17, level of significance was p < 0.05. Data from a 14-year period (2001–2014) from four universities were analyzed. Data for international students were excluded.

### Ethical consideration

The Makerere University School of Medicine Ethics and Research Committee approved this study (REC REF No. 2013-057).

## Results

In all, 7,023 students were admitted to the health professional training programs available (see list in Methods above). Overall male-to-female ratio was 2.3:1; the highest was with Gulu University (5.6:1; see Table 1). There was a significant increase in enrollment over the two time periods (p = 0.018; see Table 1).

**Table 1:** Representation by Gender, Region, and District, Admissions Data for the Two Periods, 2001–2010 and 2011–2014, and Uganda, 2001–2014.

University		Makerere		MUST	Gulu		KIU					
	
Gender M:F		1.9:1		1.8:1	5.6:1		2.4:1					
**Region**									**Total**			
	
Central		1132		416	50		110		849			
Western		515		462	57		79		867			
Eastern		483		192	27		34		846			
Northern		381		211	208		43		475			
**Gender ratio**												
	
2001–2010		1.9:1		1.8:1	5.6:1		2.4:1					
2011–2014		2:1		2.2:1	3:1		2.4:1					
Variance		↑		↑	↓		–					
**Top schools enrollment for universities**												
	
2001–2010		1055		448	29		34		156.6			
2011–2014		344		292	27		48		186.3			
p-value		0.731		0.409	0.186		0.038					
**Regional representation of students enrolled to universities in MEPI consortium**												
**Period**	**Central**			**Western**		**Eastern**				**Nortdern**		**Total**

	**No.**		**%**	**No.**	**%**	**No.**		**%**		**No.**	**%**	

2001–2010	1708		38.8	1113	25.3	736		16.7		846	19.2	**4403**
2011–2014	849		32.4	867	33.1	846		16.4		475	18.1	**2620**
P-value	<0.713			0.253		0.063				0.567		**0.018**

### Regional representation

Most students admitted were from the Central region of the country; the capital city, (Kampala), Wakiso and Mukono districts. Between 2001–2010, 39% (1,708 out of 4,403) of admissions were from central region compared to 849 out of 2,620 (32.4%) in 2011–2014 (p = 0.713), in the same region.

**Figure 1:**
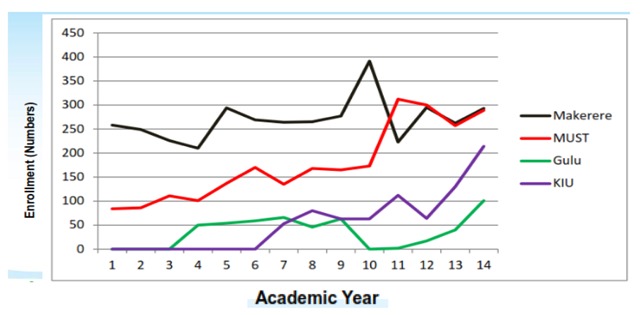
Graph Showing Universities’ Medical Education Enrollment Trends 2001–2014, Uganda.

Table 2 and Figure 1 shows admissions over the study period for all the four universities.

**Table 2:** University Admission Trend of Health Professional Students by Year, Uganda, 2001–2014.

University	2001	2002	2003	2004	2005	2006	2007	2008	2009	2010	2011	2012	2013	2014

Makerere^†^	258	249	226	210	294	269	264	265	277	391	233	295	262	243
MUST*	84	86	111	101	137	170	135	168	165	173	312	300	257	289
Gulu^++^	–	–	–	50	54	59	66	46	63	–	2	17	40	101
KIU	–	–	–	–	–	–	53	80	63	63	112	64	130	214
**Totals**	342	335	337	361	485	498	518	559	568	627	659	676	689	847

^†^ Admissions for 11 Program (MBChB, Pharmacy, Dentistry, Nursing, Radiography, Language and Speech Therapy, Dental Technology, Public Health Nursing). * Admissions for 3 Program (MBChB, Pharmacy and Nursing). ^++^ Admission for 1 Program (MBChB).

### Admissions from the top 10 schools (Table 3)

Ten secondary schools out of 2,564 (0.4%) contributed 36% (1,566 out of 4,403) of all admissions in 2001–2010 period (Table 3). All but one school (no. 6) are located in the central region of the country, where 63% (984/1566) of all student admissions were from five schools 1–5 (Table 3). Makerere University alone enrolled 1,055 out of 1,566 (67%) of the total admissions for the period 2001–2010. The same 10 schools enrolled 711 out of 2,620 (27%) students for the period 2011–2014. And Makerere University enrolled 344 out of 711 (48%) of admissions.

**Table 3:** Admissions from the 10 Top Secondary Schools by Number of Admissions, Uganda, 2001–2014.

School	University								Total No.	%
	Makerere		MUST		Gulu		KIU			

	2001–10	2011–14	2001–10	2011–14	2001–10	2011–14	2001–10	2011–14		

1. Uganda Martyrs, Namugongo	215	37	121	69	9	7	3	6	**467**	**20.5**
2. Kings College Buddo	124	31	35	25	4	2	9	2	**232**	**10.2**
3. St. Mary’s Kisubi	113	24	40	39	6	0	0	5	**227**	**10.0**
4. St. Mary Namagunga	132	37	18	11	1	3	2	0	**204**	**9.0**
5. Gayaza High School	121	34	29	19	2	2	0	4	**211**	**9.3**
6. Ntare School	63	3	86	38	0	3	0	6	**199**	**8.7**
7. Namilyango College School	78	13	42	12	5	4	3	7	**164**	**7.2**
8. Makerere College School	77	25	24	9	2	1	10	4	**152**	**6.7**
9. St. Mary’s Kitende	66	117	34	45	0	3	5	6	**276**	**12.0**
10. Kibuli Secondary School	66	23	19	25	0	2	2	8	**145**	**6.4**
**Total**	**1,055**	**344**	**448**	**292**	**29**	**27**	**34**	**48**	**2277**	

### Group discussions deliberations outputs

Disparities in admissions and access to health professional education in Uganda was stated by the discussion groups to be a major issue, and the reasons for its occurrence were identified as socio-economic, geographical and policy-driven (see **Box 1**). Other reasons included lack of sufficient household income to afford the good schools as well as other school requirements in both primary and secondary. The schools where the poor or disadvantaged have access have limited resources to run science laboratories and cannot attract and sustain experienced and well-trained teachers who demand higher wages. Career guidance is not consistently or reliably available to most students, especially in ill-equipped and under resourced schools. The rural poor who cannot access good schools are unlikely to attain high enough school academic grades to gain access to tertiary education.

The other (alternative) university entry tracks for diploma holders or mature entry have limited places, yet they may offer an opportunity for the less advantaged who would otherwise not access health professional training (HPT) or further HPT. There were calls to change strategies aimed at enhancing equity in enrollment and changing admission criteria, including introducing entry examinations among others (see **Box 1**). Entry examinations would consider a wide criterion other than only grades.

#### What should be changed

The criteria for admission should be altered in order to improve the opportunity for access; this may be easier if the available places for enrollment are increased. This has a double benefit as it increases training opportunities and improves possibility of more health workers. Since career guidance may be lacking and parents may pressure students into joining programs they have no passion for, it may be important that entry examinations (screening mechanisms) are put in place to check or test those that may not be a fit for HPT. The disadvantaged who may never have a chance to score high grades (not because they do not have the potential) should be given an opportunity for bridging courses as is done elsewhere. These bridging courses help fulfill the minimum requirements set by the training institutions. Selecting applicants outside the normally used grade-based system may face resistance. These challenges may be mitigated by involving communities e.g. to second students who should be admitted. Mechanisms on how to do this could be discussed at the community level as well as understanding the experiences gained elsewhere like in the Philippines [20].

#### How to manage the changes

The participants recommended that gathering more data to support and inform advocacy was necessary. Given the limited resources at all HPT institutions, as well as the individuals or groups that may oppose these changes, it is wise to do adequate planning and advocacy. To identify and sensitize all the major and influential stakeholders and also start small. Selecting and encouraging champions of this cause is important. A call for action in form of a policy brief was also recommended.

### Affirmative action policies

At Makerere University and in other public medical schools an extra 1.5 points were awarded to female applicants to improve their chances of joining university education. A district quota system was introduced by the government in 2004 to enable outstanding students from less privileged schools districts to acquire University education. Special schemes like talent in sports and persons with disabilities are also considered.

## Discussion

We assessed the pattern of admissions to Uganda’s medical training institutions of higher learning. We found that the amount of student enrollment overall had increased over the 14 year period. We found that there are inequalities at three levels: gender, geographical region (of origin) and secondary schools attended. Equity is considered as treating everyone the same way. Equality aims to promote fairness but it can only work if everyone starts from the same place and needs the same help. Inequality in accessing HPE is a phenomenon that is seen in sub-Saharan Africa and middle-income countries elsewhere [2,3]. In this study females had significantly less access than males. The likelihood of joining HPE was higher if one attended any of the top 10 schools located in central Uganda. These schools contributed a third of all admissions yet they represented only 0.4% of the 2,564 secondary schools in the country. In all, at least 7,000 students were admitted over a fourteen year period, yet Uganda, with close to 37 million people, has less than 5,500 registered practicing doctors, specialists and generalists combined [15]. When access to training is skewed to the elite and well-to-do, upon graduation the (graduates) are reluctant to go, stay and work in underserved and difficult to reach areas. This worsens the divide between the rich(mostly in the cities) and the rural poor, contributing to poor health outcomes [10,16].

**Box 1: Perspectives from Stakeholders for Correcting Admission Inequalities in Medical Schools in Uganda, 2013.**

**Why there are inequalities (of Gender, Region of Origin, School attended)?**

- Low household incomes
- Rural schools have limited resources such as laboratories, trained teachers
- Poor or absent career guidance
- Using grades only admission criteria
- Few training institutions and limited number of places
- Limited slots for diploma holders
- Quota system not working for science based courses

**What should we change:**

- Admission criteria; create parallel entry tracks for the disadvantaged
- Increase enrollment
- Introduce entry exams/aptitude tests
- Introduce bridging programs for the disadvantaged to match the high achievers
- Communities to participate in selection of entries via the special/parallel track
- Training institutions to relocate or increase training in community/rural sites
- Introduce credit transfer system
- Introduce multiple entry and exit routes and intermediate awards
- Career guidance for students in high school

**How do we manage the change (strategies):**

- Incremental change; ‘low hanging fruits’
- Sensitization of stakeholders: Ministry of Health, Ministry of Education and Sports, Uganda National Council of Science & Technology, Professional bodies (Uganda Medical Association, Nursing council, Pharmacists Council, Dental Council, Allied Professionals), School head teachers
- Advocacy for policy change (policy briefs, create translational fora)
- Individual schools to engage their University Senates and Councils
- Gather more supporting data e.g. Socio economic status of parents, work station deployment trends

### Bottlenecks to access

For the most part student admissions and are directly admitted from high school, and are largely based on academic scores. To achieve high enough grades a candidate should have studied in a highly resourced secondary school. Attendance at any of these schools requires that the parents or guardians are able to pay the required school fees and buy scholastic materials that may not be provided at the school. To study at a school good enough to facilitate passing examinations with high enough grades to be admitted to the few, better institutions of higher learning is a daunting task for an average Ugandan citizen. Earlier on in primary schools, the lack of or inadequate food at school leads to increased school drop outs, which is common in rural schools. Entry into health professional training institutions may be via diploma and mature entry tracks. Diplomas holders (or the equivalent) access admission through the diploma track. Mature age is defined as at least 25 years of age, and a mature entrant must pass an entrance examination; however, only those with the highest scores are admitted.

These diploma holders are professionals in a health related field such as physician assistants (clinical officers) or bachelorette nurses. The latter has fewer places and therefore fierce competition.

### What should change

The Joint Admission Board (JAB), a body in charge of university admissions, largely uses high school grades, to determine who gets admitted for university education. Using grades is objective, transparent and easy to manage. There have been efforts to improve or modify these criteria; a clear example was an attempt to deal with gender disparities by introducing an affirmative action policy. In the case of Makerere University, an extra 1.5 points were awarded to female applicants [17,18,19]. This seemed to improve access, as the ratio of male: female improved in favour of female students, but these are downstream measures. Upstream measures such as improving access at secondary level for science subjects for the girls and rural children need to be consolidated.

There is anecdotal information that some parents force their children to take up courses or careers that their children are not interested in, and upon graduation they hand over the degree certificates to their parents and go off to embark on careers of their interest (non-medical careers in this case), so providing a mechanism to filter those without interest in the medical profession would be helpful. The screening should detect a willingness to serve the disadvantaged. This could be done by introducing entry exams where these aspects can be assessed. An innovative approach to include the voice of underserved communities in deciding who among the applicants should be seconded for training was suggested in the group discussions. These could be high school students who come from these communities or health workers already working in those communities but wishing to upgrade.

### How to manage the change

In this paper, we further highlight the perspectives of a group of stakeholders on how these changes of admission criteria could be tackled (**Box 1**). Two entry tracks were proposed. First, the voice of underserved communities in selecting trainees who would be given priority. Disadvantaged communities should be identified and offered a number of places which they should fill until their health worker ratio improves. Second, an increment of places for high academic achievers was proposed as well as establishing the minimum requirements of admission to University. Other considerations should be made such as introducing bridging (remedial) courses. The idea of communities participating in the selection of trainers was novel and is already being used in the Philippines [20]. It may involve already existing platforms, e.g. district health service committees and local councils. This may ensure that whoever is selected will go back to that community to serve as a way to give back.

Small, incremental changes were proposed. This is a practical approach cognizant of the fact that major changes will require large resources like more teachers, space and materials. The introduction of entry examinations or aptitude tests is meant to screen for genuine intention and calling to practice medicine, as opposed to seeking only prestige and money. Institutions in many countries have practiced this approach. These changes could be coupled with systematic career guidance sessions in schools so that students make fairly informed choices.

### Bridging programs

Bridging courses are aimed at assessing skill and strengthening a candidate’s medical foundation, especially for those whose grades were below the minimum required. This can also be used to build on non-academic requirements like communication, use of technology and problem solving skills. Bridging programs may be helpful for those who may not have attained the academic grades and may not be very fluent in the language of instruction (English). Similar programs have been carried out in the Republic of South Africa to overcome racial segregation/apartheid effects [21].

The introduction of credit transfer systems allows practicing professionals to gain access without having to repeat some courses done during prior training. Various methods to sensitize all stakeholders were suggested including policy briefs, scientific publications and gathering more supporting data.

### Aptitude tests

These are academic and nonacademic requirements applicants are expected to have. The academic requirements are subjects completed with minimum grades achieved. Disadvantaged students may be assisted in the catch-up phase by taking them through bridging programs. They are then subjected to entry tests prior to enrollment in the desired health professional program of study. The non-academic requirements are related to what the practice of medicine demands, such as time management, teamwork, and decision-making. These are part of the professionalism expected of trainees by the profession and the community they will serve.

### Career guidance

Although wide ranges of specialty options now exist after qualification, nevertheless making such career choices in medicine and related disciplines, particularly at a young age, is daunting for many. The training is mostly extensive, requires dedication and lifelong learning. Career guidance is therefore critical to avoid non-completion or abandonment of the profession after completing the arduous training and obtaining a qualification or soon after.

## Limitations

The JAB records had missing data; for example, we were not able to record parental occupation (a proxy to socio-economic status). We inadvertently did not include school head teachers among the group discussions (stakeholders) thus their views were missed. It is possible that some of the responses regarding student origin may not have been truthful as some students raised in cities may have provided misleading information that they come from disadvantaged communities.

## Conclusions

There were significant inequalities for higher education training in Uganda, by gender, regional representation and school attended. Modifying the admission criteria and increasing enrollment may reduce on these inequalities.

## Figures and Tables

**Table:** 

JAB	Joint Admissions Board
MESAU	Medical Education for Equitable Services to all Ugandans
MEPI	Medical Education partnership Initiative
KIU	Kampala International University
HPE	Health Professional Education
PUJAB	Public University Joint Admissions Board
